# Endometriosis and sexual disorders: the effect of surgical and medical treatment, a multicentre cross-sectional study

**DOI:** 10.12688/f1000research.141537.1

**Published:** 2023-10-31

**Authors:** Tommaso Capezzuoli, Elisa Maseroli, Fabio Barra, Silvia Vannuccini, Linda Vignozzi, Paola De Mitri, Silvia Baggio, Marcello Ceccaroni, Felice Petraglia

**Affiliations:** 1 Department of Experimental, Clinical and Biomedical Sciences, Division of Obstetrics and Gynecology, Careggi University Hospital, University of Florence, Florence, Tuscany, Italy; 2 Andrology, Women's Endocrinology and Gender Incongruence Unit, Careggi University Hospital, University of Florence, Florence, Tuscany, Italy; 3 Department of Health Sciences (DISSAL), University of Genoa, Genoa, Liguria, Italy; 4 Unit of Obstetrics and Gynecology, P.O. "Ospedale del Tigullio"- ASL4, Genoa, Italy; 5 Department of Experimental and Clinical Biomedical Sciences "Mario Serio", University of Florence, Florence, Tuscany, Italy; 6 Department of Obstetrics and Gynecology, Gynecology Oncology and Minimally-Invasive Pelvic Surgery, International School of Surgical Anatomy (ISSA), IRCCS Ospedale Sacro Cuore Don Calabria, Verona, Italy

**Keywords:** Endometriosis, sexual health, hormonal treatment, surgical treatment, female sexual dysfunction

## Abstract

**Background:**

Sexual health is a major concern in women with endometriosis, however only a few controlled studies have examined this with validated instruments. The effect of hormonal treatments on sexual function in endometriosis is also an underrated topic. The aim of this study was to investigate sexual function of patients with endometriosis by a specific tool to better evaluate their sexual function (including different domains), and the influence of hormonal treatment or surgery on these parameters.

**Methods:**

An observational, cross-sectional, multicentre study was conducted in a group (n=194) of sexually active, women aged 25–45 years old, with surgical or ultrasonographic diagnosis of endometriosis, referred to the Endometriosis Center of Careggi University Hospital or Negrar di Valpolicella. Sexual function was assessed by administering the Female Sexual Function Index (FSFI), which assesses the domains of desire, arousal, lubrication, orgasm, satisfaction and pain. FSFI scores were compared to those of a control group (n=58) and according to the treatment received by patients with endometriosis.

**Results:**

Ovarian endometriosis was present in 50 patients (25.8%), deep infiltrating endometriosis in 65 patients (33.5%) and both in 79 patients (40.7%). Adenomyosis coexisted in 102 patients (52.6%). Women with endometriosis reported a mean total FSFI score of 18.3 [4.2-25.8] (< 26.55), indicating female sexual dysfunction (FSD) in all patients. At multivariate analysis, after adjusting for confounders (BMI and hormonal therapy), women with endometriosis presented significantly lower scores than controls in all the FSFI (p<0.001). Patients with endometriosis under hormonal treatments (n=124; 64%), regardless of the type, had significantly lower scores in all FSFI subscales and total score, even after adjusting for confounders—age, BMI and history of surgery.

**Conclusions:**

Patients with endometriosis are at risk for FSD, encompassing not only dyspareunia, but all domains of sexual function. Hormonal treatments do not result in improvement in sexual symptoms.

## Introduction

Endometriosis is a benign chronic inflammatory disease defined as the presence of endometrium outside of the uterine cavity, and the most recognized mechanisms that explain the ectopic location of the endometrial cells are retrograde menstruation and stem cell differentiation. ^1^ The disease affects 10% of women at reproductive age and it is characterized by painful symptoms and infertility, affecting quality of life (QoL). ^2^^–^^5^

Considering that patients with endometriosis are young and sexually active, sexual health is a major concern and should be carefully evaluated. ^6^ In fact, endometriosis is associated with reduced psychological well-being, and impaired sexual life and relationships. Patients often complain about dyspareunia, decreased sexual satisfaction, painful sex and overall altered sexual functioning, ^7^ driving to an impaired quality of sexual life with a significant negative effect on their relationships. ^8^^–^^11^ Besides, the coexistence of adenomyosis is associated with a worse quality of sexual life than only endometriosis. ^12^^,^^13^

Moreover, multiple surgery and long-term hormonal treatments may represent additional contributing factors to negative sexual function in endometriosis. Induced transient menopausal state associated with some hormonal treatments, such as gonadotropin releasing hormone analogues (GnRHa), affects brain areas involved in sexual response. Also combined oral contraceptives (COCs) ^14^ and progestin treatment may induce a change in sexual functioning. ^15^ In fact, despite medical treatment having a role in endometriosis management given its efficacy on painful symptoms ( *e.g.*, dyspareunia) and prevention of disease recurrence and progression, it may influence the sexual well-being. ^7^

The aim of the present study was to investigate sexual function of patients with endometriosis by a specific tool to better evaluate their sexual function (including different domains, *i.e.*, desire, arousal, lubrification, orgasm, satisfaction and pain), and the influence of hormonal treatment or surgery on these parameters.

## Methods

### Ethical considerations

The study was approved by the local Institutional Review Board (Comitato Etico Regione Toscana-Area Vasta Centro-CEAVC, n. 14558_oss approved on 28 May 2019), and all participants provided written informed consent to be included in the series.

### Study design

An observational cross-sectional multicentre study was conducted in a group (n=194) of fertile age women (25–45 years old) with endometriosis, consecutively referred to two different hospitals, both reference Centres for Endometriosis (Careggi University Hospital, Florence and Negrar di Valpolicella, Verona, Italy) and recruited between January 2022 and February 2023.

### Participants

The study group was constituted of patients with surgical or imaging diagnosis of endometriosis (mean age was 35.45 ± 7.44 years). Ovarian endometriosis (OMA) was present in 50 patients (25.8%), deep infiltrating endometriosis (DIE) in 65 patients (33.5%) and both in 79 patients (40.7%). Adenomyosis coexisted also in 102 patients (52.6%). Previous surgery for endometriosis was performed by 24.2% (n=47) patients: 37 (78.7%) underwent one previous surgery, whereas 21.3% more than two surgeries for endometriosis. Regarding medical treatments, 64% (n=124) were under a hormonal therapy. The majority of patients were treated with dienogest (59.7%, n=74), followed by those treated with COCs (21%, n=26), desogestrel (7.3%, n=9), GnRHa (4%, n=5), norethisterone acetate (NETA) (3.2%, n=4), drospirenone (2.4%, n=2) and levonorgestrel intrauterine system (LNG-IUS) (2.4%, n=3).

A group of women without endometriosis attending the two Women’s Endocrinology outpatient clinics were enrolled as the control group (n= 58) (mean age was 34.55 ± 8.76 years). Controls were consulting for contraceptive needs, or for follow-up for thyroid or metabolic disorders. The abovementioned endocrinologic conditions were clinically stable since at least six months, respondent to treatment and presenting laboratory or imaging parameters within the normal ranges. These were the criteria to be eligible as controls in the study. Data were collected by an extensive review of clinical records of patients in the follow-up of these outpatient clinics.

Exclusion criteria were: menopausal status, pregnancy, desire of pregnancy when the survey was conducted or previously attempts to conceive, both naturally or through assisted reproductive technologies, breastfeeding, systemic diseases—including previous or active cancer, polycystic ovary syndrome, hyperandrogenism, hyperprolactinemia, uncontrolled psychiatric diseases, alcohol or drug abuse, and use of medications with a possible influence on sexual function except for hormonal contraception ( *i.e.*, antidepressant and anxiolytic drugs). The ability to provide written informed consent and having engaged in sexual activity in the previous month were considered as inclusion criteria.

During the follow-up visit, patients were interviewed through:

(i) a structured questionnaire containing all clinical information regarding the history of the patient (in particular age, body mass index (BMI), parity and current use of hormonal treatment). The hormonal treatments used were: progestins, GnRHa or continuous COCs, for a minimum of 12 months;
(ii) a structured questionnaire containing all clinical information about female sexual function. Sexual symptoms were investigated by using the gold standard tool for the screening of Female Sexual Dysfunction (FSD), the Female Sexual Function Index (FSFI). This self-administered questionnaire analyses overall levels of sexual function and its primary components: sexual desire, arousal, lubrication, orgasm, pain, and satisfaction. ^16^ The FSFI is composed by items with answers codified on a 5-point Likert scale ranging from 1 to 5, with higher scores indicating greater levels of sexual functioning for each item. The total score, resulting from the sum of the five domains, ranges from 2 to 36; a total score of 26.55 has been found to provide an excellent cut-off to distinguish women with and without FSD. ^17^

All participants filled out the answers to the FSFI questionnaire themselves, whereas baseline and medical data were asked by a healthcare professional.

### Statistical analysis

Data were reported as mean ± SD when normally distributed, as median (quartiles) when non-normally distributed and as percentage and number when categorical. The unpaired 2-sided Student’s t-test and the Mann-Whitney U test were applied for the assessment of between-group differences, whenever appropriate. Multivariate models, with adjustment for relevant clinical confounders, were conducted by means of analysis of covariance. Statistical analyses were performed in SPSS 26.0 IBM SPSS Statistics (RRID:SCR_016479) for Windows (SPSS Inc, Chicago, IL, USA).

## Results

Cases and controls were similar for age, whereas those with endometriosis showed a lower BMI than controls (22.55 ± 3.81 *vs.* 24.74 ± 6.93, p=0.002) and were more likely to use hormonal therapy (64% *vs.* 19%, p<0.001). In terms of sexual function, women with endometriosis reported a mean total FSFI score of 18.3 [4.2-25.8] (< 26.55), indicating FSD in all patients. Conversely, those in the control group obtained a mean total score of 32.0 [28.8-33.4], thus excluding FSD. Comparing each FSFI domain, at univariate analysis, women with endometriosis presented significantly lower scores than controls, in desire, arousal, lubrication, orgasm, satisfaction and pain (p<0.001) ( Table 1), indicating sexual functioning impairment.

**Table 1.** Differences between women with endometriosis and controls.

Patient data and FSFI domains	Endometriosis (n=194)	Controls (n=58)	p	p (adjusted)
**Age (years)**	35.45 ± 7.44	34.55 ± 8.76	0.219	
**BMI (kg/m2)**	22.55 ± 3.81	24.74 ± 6.93	0.002 *	
**Parity (n, %)**	68 (35%)	17 (29%)	0.417	
**Hormonal therapy (n, %)**	124 (64%)	11 (19%)	<0.001 *	
**FSFI Desire**	2.4 [1.8-3.6]	4.2 [3.6-4.8]	<0.001 *	<0.001 *
**FSFI Arousal**	3.3 [0.3-4.5]	5.4 [4.8-5.7]	<0.001 *	<0.001 *
**FSFI Lubrication**	3.6 [0.0-5.4]	5.8 [5.4-6.0]	<0.001 *	<0.001 *
**FSFI Orgasm**	3.2 [0.0-4.8]	5.6 [5.1-6.0]	<0.001 *	<0.001 *
**FSFI Satisfaction**	3.6 [0.4-5.2]	5.6 [5.2-6.0]	<0.001 *	<0.001 *
**FSFI Pain**	2.0 [0.0-3.6]	6.0 [5.5-6.0]	<0.001 *	<0.001 *
**FSFI Total**	18.3 [4.2-25.8]	32.0 [28.8-33.4]	<0.001 *	<0.001 *

Multivariate analysis was adjusted for age, BMI and use of hormonal therapy. The symbol * indicates statistically significant difference. BMI = body mass index. FSFI = Female Sexual Function Index. HT = hormonal therapy. Data were reported as mean ± SD when normally distributed, as median (quartiles) when non-normally distributed and as percentage and number when categorical. Multivariate models, with adjustment for relevant clinical confounders, were conducted by means of analysis of covariance.

At multivariate analysis, after adjusting for confounding factors (BMI and use of hormonal therapy), all the reported differences between the two groups retained statistical significance (p<0.001 for all the scores; Table 1).

In a second step, sexual function in patients with endometriosis was further investigated, exploring the differences between those taking (64%, n=124) and not taking (36%, n=70) hormonal treatment (GnRHa, progestins, oral contraceptives) and the potential influence of previous surgery for endometriosis. Hormonal therapies, regardless of the type, were associated with significantly lower scores in all FSFI subscales and in total score, indicating worse sexual functioning. After adjusting for confounders—age, BMI and history of surgery—all the differences retained statistical significance ( Table 2).

**Table 2.** Differences in FSFI scores between women with endometriosis taking and not taking hormonal therapy.

FSFI domains	Hormonal therapy (n=124)	No hormonal therapy (n=70)	p	p (adjusted)
**FSFI Desire**	2.4 [1.8-3.6]	3.0 [2.4-3.6]	0.045 *	0.016 *
**FSFI Arousal**	2.5 [0.0-4.2]	3.9 [2.1-4.9]	0.010 *	0.002 *
**FSFI Lubrication**	3.3 [0.0-5.4]	4.0 [2.4-5.7]	0.028 *	0.010 *
**FSFI Orgasm**	2.4 [0.0-5.4]	4.4 [1.6-5.6]	0.003 *	<0.001 *
**FSFI Satisfaction**	3.2 [0.0-4.8]	4.8 [2.4-5.6]	0.002 *	<0.001 *
**FSFI Pain**	1.6 [0.0-3.2]	3.6 [1.2-4.8]	<0.001 *	<0.001 *
**FSFI Total**	17.2 [3.6-24.5]	21.9 [13.0-28.0]	<0.001 *	<0.001 *

Multivariate analysis was adjusted for age, body mass index, and a history of previous surgery for endometriosis. The symbol * indicates statistically significant difference. indicates statistically significant difference. FSFI = Female Sexual Function Index. Multivariate models, with adjustment for relevant clinical confounders, were conducted by means of analysis of covariance.

## Discussion

The present study confirmed that patients with endometriosis have worse sexual function compared to healthy controls in all FSFI domains (desire, arousal, lubrification, orgasm, satisfaction and pain) and FSFI total score.

Our results confirmed those of a recent meta-analysis ^18^ showing that patients with endometriosis have lower scores of FSFI total score with poor sexual function. Considering that endometriosis is a disease that affects sexually active young women, the evaluation of sexual function in the context of patient’s global management is crucial in order to improve the global QoL. ^7^ In fact, endometriosis is not only characterized by sexual pain, but they also present an important impairment in several domains of sexuality (desire, arousal, lubrification, orgasm, satisfaction). A recent study showed patients with endometriosis have a worse score in the short-form of McGill Pain Questionnaire (SF-MPQ), pain subscale of FSFI, and Sexual Distress Scale (FSDS). ^19^ Furthermore, they reported more negative emotions toward sexuality and seem to be characterized by an impairment in body image, ^20^ depressive symptoms, ^21^ worse health related QoL (HRQoL) and unemployed work status. ^22^

Several mechanisms play a role in increasing the risk of FSD in women with endometriosis. First, in women with dyspareunia, fear and anticipation of pain strongly affect the global sexual response, thus increasing sexual inhibition and reducing spontaneous desire and sexual fantasy. In addition, psychological and interpersonal correlates of endometriosis, including fertility issues, low self-esteem, and body image concerns, contribute to negatively affect sexual function in its different areas. ^7^ In recent years, evidence is accumulating that indicates a relevant overlap between endometriosis and superficial dyspareunia, and a high prevalence of Genito-pelvic pain and penetration disorder (GPPPD) in endometriosis. ^23^ These comorbidities are also likely to play a role in compromising the sexual experience of affected women.

Considering that endometriosis is a chronic condition, medical treatment is the primary choice for improving symptoms, preventing or treating recurrences and planning surgery or ART. GnRH analogues or antagonists, progestins, combined oral contraceptives block cyclic menstruation and reduce endometriosis-related pain. ^1^^,^^5^ The present study showed that hormonal treatment, regardless of the type, is not associated with an improvement in sexual function, showing lower scores in all FSFI subscales and total score. After adjusting for confounders—age, BMI and history of surgery—all the differences retained statistical significance.

The strong hypoestrogenic effect of GnRH agonists seems to be associated with a significant decline in libido and vaginal lubrication. ^6^ Despite the use oral contraceptives and progestins in healthy women was previously described to be associated with negative sexual side effects (sexual activity, arousal, pleasure and orgasm and more difficulty with lubrication), ^6^^,^^14^ a recent observational study detected an improvement of sexual quality of life in patients with DIE with or without adenomyosis after 12 months of treatment with a combined oral contraceptive (2 mg dienogest/30 μg ethinyl oestradiol). ^13^

Considering the effect of surgical treatment, it is an option that works on pain relief and improvement of quality of sex life in symptomatic women with endometriosis. ^24^^,^^25^ On the other hand, persistent or recurrent endometriosis after unsuccessful first-line conservative surgery is associated with severe deep dyspareunia and low FSFI score, below the cut-off for normal sexual function. ^6^^,^^26^ Furthermore, the comparison between surgical treatment *versus* a low-dose progestin therapy among patients with deep dyspareunia shows an immediate significant improvement of pain after surgery, but recurrent over time; on the contrary, those on low dose of NETA have a slight decrease in dyspareunia, though progressively declining. ^26^ Medical and surgical treatment should be carefully evaluated because they often do not consistently allow for the global improvement of sexual function, despite their strong effect on painful symptoms of affected patients.

Finally, endometriosis is frequently associated with gynaecological and systemic comorbidities that may cause sexual dysfunction and impair the Qol. ^11^^,^^27^^–^^29^ Adenomyosis determines further a high rate of altered sexual function in patients with endometriosis. ^12^^,^^13^ Autoimmune, inflammatory, psychiatric and neurological disorders are commonly described in patients with endometriosis ^30^^,^^31^ and have a strong effect on global QoL and also sexuality. Therefore, the evaluation of eventual gynaecological and systemic comorbidities is mandatory in patients with endometriosis.

The present study has some limitations. First, sexual-related distress was not assessed, and this is a relevant aspect of sexual dysfunction. Second, all women were sexually active, but the relational component ( *i.e.*, the presence of a stable relationship, couple conflicts, sexual dysfunction in the male partner) was not evaluated. Furthermore, our study population is a selected sample of patients with severe endometriosis referred to highly specialized centres and most likely include cases with recurrent symptoms after either surgical or hormonal treatment.

In conclusion, sexual dysfunction is a common finding in patients with endometriosis and a multidisciplinary approach, including a psychological support and the contribution of other specialists for systemic comorbidities, is warranted. ^7^ In fact, the traditional hormonal or surgical management do not significantly improve such as an important aspect as sexual function, and a multimodal approach is required.

## Data Availability

To protect the patients’ privacy the present study data access was restricted. The anonymous data about patients’ sexual function can be shared with readers and reviewers. To apply for access to the data, readers or reviewers can contact Dr. Tommaso Capezzuoli ( tommasocapezzuoli@unifi.it ). While applying for access, reader or reviewer should give a signed letter mentioning that they will not share the data with a third party and it will used only for academic purpose.
