# Supplementary material for: Endometriosis and sexual disorders: the effect of surgical and medical treatment, a multicentre cross-sectional study
Source: F1000Res. 2023 Oct 31;12:1424. [Version 1] doi: 10.12688/f1000research.141537.1 (PMC11240085; doi:10.12688/f1000research.141537.1)
Supplement: Peer review history

# Reviewer report: Reviewer response for version 1

**DOI:** 10.5256/f1000research.154993.r285939

**Published:** 2024-07-11

**Authors:** Giussy Barbara

**Affiliations:** 1 University of Milan, Milan, Italy

I read with interest the article by Capezzuoli et al. entitled "Endometriosis and sexual disorders: the effect of surgical and medical treatment, a multicenter cross-sectional study".

 The authors conducted a multicenter cross-sectional study to evaluate sexual function in women with and without endometriosis using the FSFI.

 Several studies have already investigated the impact of endometriosis on sexual function, and it is well known that endometriosis has a negative impact on female sexuality. However, in this study, the authors found that patients with endometriosis under hormonal treatment (n=124; 64%), regardless of type, had significantly lower scores on all FSFI subscales and total score, even after adjusting for confounders - age, BMI, and history of surgery. This is a somewhat novel finding and warrants further investigation.

 However, so far studies have suggested that medical treatment of endometriosis can reduce pain during intercourse and consequently improve sexuality.

 However, female sexuality is a very complex dimension, influenced not only by pain but also by several other potential factors, both psychological and social and relational.

 My suggestion to the authors is to discuss this point in the paper as a limitation of the results, since they did not have an investigation of psychological, social and relational factors, and this could have had an impact on the results.

 Overall, the paper is well written and the discussion is logically supported by the results.

 I recommend indexing.

Is the work clearly and accurately presented and does it cite the current literature?

Yes

If applicable, is the statistical analysis and its interpretation appropriate?

Yes

Are all the source data underlying the results available to ensure full reproducibility?

Yes

Is the study design appropriate and is the work technically sound?

Yes

Are the conclusions drawn adequately supported by the results?

Yes

Are sufficient details of methods and analysis provided to allow replication by others?

Yes

Reviewer Expertise:

gynecology, endometriosis, sexual health

I confirm that I have read this submission and believe that I have an appropriate level of expertise to confirm that it is of an acceptable scientific standard.

# Reviewer report: Reviewer response for version 1

**DOI:** 10.5256/f1000research.154993.r231806

**Published:** 2024-02-21

**Authors:** Maria Grazia Porpora

**Affiliations:** 1 Department of Maternal and Child Health and Urology, “Sapienza” University,, Rome, Italy

I have carefully read the manuscript entitled *Endometriosis and sexual disorders: the effect of surgical and medical treatment, a multicentre cross-sectional study,* by Tommaso Capezzuoli, Elisa Maseroli, Fabio Barra, Silvia Vannuccini, Linda Vignozzi, Paola De Mitri,  Silvia Baggio, Marcello Ceccaroni and Felice Petraglia. 

 The study aimed to analyze del sexual functioning in women with endometriosis and /or adenomyosis. In this multicentric study, all women with surgical or sonographic diagnosis of endometriosis or adenomyosis answered to a structured questionnaire containing all clinical information on their medical history and to the FSFI questionnaire which is validated for the evaluation of the sexual function. Results were compared with those of a control group.

 The authors found that all affected women, independently from the type of lesions, had a lower FSFI score than controls, indicating FSD in all patients. Women taking hormonal therapies had worse results in terms of FSD.

 The paper is interesting and well written, but I have a few comments for the Authors:

 1. Surgery, particularly when involving the bowel and its function, can worsen the self- body image perception and the presence of bowel problems significantly affects sexual functioning (Boyd T, et al 2022.) [Ref 1]; did you find any difference between those who had undergone surgery and those who received only medical treatments? In addition, did you find differences in sexual function according to the type of surgery?

 2. Surprisingly, in this study, all kinds of medical therapy worsened the sexual functioning of patients. These results can be easily justified in women taking GnRh analogues or even oral progestins but the detrimental effects on sexual functions are less clear in women taking oral contraceptives. Do you think that the lack of difference between treatments could be related to the small number of patients in each group? Could it be possible that a long history of disease may have affected the quality of life and have determined psychological problems, thus influencing the results?

 3. Psychological factors and mood disorders were not evaluated in this paper, but they may have influenced the results.  Please make a comment about this.    

 4. It is also possible that marital status and a stable affective relationship can improve the patient’s sexual life as observed by Giuliani et al. in 2016 (Giuliani et al. 2016)[Ref2]. Could you please make a comment on this aspect?”

Is the work clearly and accurately presented and does it cite the current literature?

Yes

If applicable, is the statistical analysis and its interpretation appropriate?

Yes

Are all the source data underlying the results available to ensure full reproducibility?

Yes

Is the study design appropriate and is the work technically sound?

Yes

Are the conclusions drawn adequately supported by the results?

Yes

Are sufficient details of methods and analysis provided to allow replication by others?

Yes

Reviewer Expertise:

endometriosis, adenomyosis, pelvic pain, minimally invasive surgery

I confirm that I have read this submission and believe that I have an appropriate level of expertise to confirm that it is of an acceptable scientific standard.

## References

[1] : Sexual Dysfunction in Female Patients with Inflammatory Bowel Disease: An Overview. *Clin Exp Gastroenterol* .2022;15: 10.2147/CEG.S359367 213-224 10.2147/CEG.S359367 36540885 PMC9759977

[2] : Quality of life and sexual satisfaction in women suffering from endometriosis: An Italian preliminary study. *Sexologies* .2016;25(1) : 10.1016/j.sexol.2015.03.004 e12-e19 10.1016/j.sexol.2015.03.004
